# Motor imagery enhances core training effects on lumbar proprioception in elite swimmers: a randomized controlled trial

**DOI:** 10.3389/fphys.2025.1667536

**Published:** 2025-09-01

**Authors:** Mónica Solana-Tramunt, Ana Bofill-Ródenas

**Affiliations:** ^1^ Facultat de Psicologia, Ciències de l’Educació i de l’Esport Blanquerna, Universitat Ramon Llull, Barcelona, Spain; ^2^ INEFC-Barcelona Research Group on Sport Sciences (GRCEIB). Institut Nacional d’Educació Física de Catalunya, Universitat de Barcelona, Barcelona, Spain

**Keywords:** lumbar proprioception, undulatory underwater swimming, high performance, conscious movement, lumbar motor control, core control, core training

## Abstract

Lumbar proprioception is essential for executing effective movements during the undulatory underwater swimming (UUS) technique. Core exercises are commonly used to strengthen the musculature involved in UUS, and variations in sensory input and attentional focus may modulate neuromuscular responses, influencing training outcomes. This study investigated the impact of repeated maximal lumbar movements performed with closed eyes and motor imagery on lumbar proprioception in elite swimmers, compared to performing the same exercises with proper technique and controlled breathing alone. Methods: A total of 57 elite swimmers (34 males, 20.2 ± 4.2 years; 23 females, 20.7 ± 3.3 years) volunteered and completed the study. Participants were randomly assigned to two experimental groups (EG1 and EG2) and one control group (CG). All groups followed the same swimming and physical training program, with EG1 and EG2 completing additional lumbar flexion-extension exercises. Both EG1 and EG2 performed three sets of 10 repetitions at a controlled breathing pace, 6 days per week for 11 weeks. EG1 completed the exercises with eyes closed and motor imagery; EG2 performed the same exercises focusing only on technique. Lumbar joint position sense in the sagittal plane was assessed using an electrogoniometer while seated on a Swiss ball. Results: Significant group differences were found for relative repositioning error (REr) (F = 6.75, p = 0.012) and absolute error (ABSEr) (F = 7.34, p = 0.009). EG1 showed large positive effect sizes (REr d = 0.91; ABSEr d = 1.24), EG2 showed negative effects (REr d = −0.56; ABSEr d = −1.17), and CG had no meaningful changes. Conclusion: Motor imagery enhances proprioceptive accuracy beyond physical training alone. Trial Registration: NCT06747702.

## 1 Introduction

Core control exercises are widely recognized for enhancing postural stability, movement efficiency, and injury prevention in both athletic and rehabilitative contexts. These exercises engage both deep and superficial core muscles—such as the transversus abdominis, multifidus, and obliques—which are critical for spinal alignment and force generation during dynamic tasks (Huang et al., 2025). Although core strengthening benefits postural control, the underlying physiological mechanisms remain under investigation, especially regarding resistance training’s influence on the neurological properties of lumbar musculature (Solana-Tramunt et al., 2025).

Conscious lumbar control is highly dependent on proprioceptive input (Georgy, 2011), yet the lumbar spine remains under-investigated in athletic populations despite its importance for limb efficiency, trunk stability, and postural balance (Wang et al., 2016; Han et al., 2014). Sensorimotor control may be more relevant than strength or endurance for achieving lumbar stability and mobility. Most studies focus on sagittal plane movements due to their link with low back pain and injury risk (Crasto et al., 2019; Warneke et al., 2024; Solana-Tramunt et al., 2019a). Lumbar proprioception is often assessed through joint position sense (JPS), typically measured by joint angle recognition accuracy using absolute error (AEr) during active or passive tasks (Hillier, 2015).

Effective movement acquisition relies on proprioceptive feedback (Steinberg et al., 2016; Han et al., 2013). While novice athletes depend on conscious control and closed-loop feedback, experienced athletes use sensory monitoring more selectively (Bachmann and Francis, 2013). Core stability is a central focus in most strength and conditioning programs for elite swimmers, as core muscle engagement during swimming has been proposed to reduce form resistance and drag, thereby enhancing swimming speed (Khiyami et al., 2022; Karpiński et al., 2020; Ji et al., 2021). Despite this, the lumbar spine is known to have limited afferent and motor cortical representation in both somatosensory and motor areas (Mok Nicola WNW Department of Rehabilitation Sciences THKP, 2013). Furthermore, maintaining posture, balance, and alignment in the water is considered essential for optimizing propulsion and minimizing drag during swimming performance (Puel et al., 2010; Houel et al., 2010).

For elite swimmers, core stability is essential for reducing drag and enhancing hydrodynamics during swimming, particularly in the underwater phase following starts and turns. These underwater sections, where swimmers maintain higher velocities than surface swimming due to reduced drag, are crucial for performance—making undulatory underwater swimming (UUS) a key focus for competitive swimmers (Ruiz-Navarro et al., 2022; Matsuura et al., 2020; Veiga et al., 2022). Recent studies have identified lumbar spine mobility, core stiffness, and the ability to control high-speed lumbar flexion-extension as key contributors to effective UUS (Ruiz-Navarro et al., 2022; Veiga et al., 2022). UUS requires alternating trunk, hip, and leg flexor/extensor muscle contractions, with trunk and pelvic coordination driving propulsion. Greater lumbar range of motion (ROM), particularly in flexion-extension, has been linked to higher velocities during dolphin kicking (Matsuura et al., 2020; Veiga et al., 2022). Electromyographic analyses have highlighted muscle synergies—especially involving the internal oblique, rectus abdominis, erector spinae, and multifidus—that facilitate effective pelvic tilt during the dolphin kick. Lumbar proprioception, therefore, plays a neuromechanical role in modulating core-driven undulatory movement pattern (Matsuura et al., 2020).

Evidence suggests that elite swimmers demonstrate superior lumbar proprioceptive acuity—i.e., lower relative repositioning error (REr)—compared to recreationally active individuals (Solana-Tramunt et al., 2019b). Enhanced lumbar JPS may also contribute to injury prevention by refining movement accuracy and reducing strain during high-speed repetitions (Hsu et al., 2024). Sensory input—particularly vision and proprioception—strongly influences motor control during core stabilization exercises. When vision is removed (e.g., eyes closed), reliance on proprioceptive feedback increases, especially from muscle spindles (Blanchard et al., 2013; Nieto-Guisado et al., 2022). This sensory shift may improve neuromuscular coordination and proprioceptive acuity. Moreover, consciously directing attention to core muscle activation has been shown to enhance motor learning and muscle recruitment, with implications for both rehabilitation and performance training (Okada et al., 2011; Shamsi et al., 2015; Cabre et al., 2022).

Core and mobility training are fundamental in dryland programs for swimmers, supporting the biomechanical demands of aquatic movement (Zhou et al., 2024). However, the influence of sensory and attentional modulation during core exercises remains understudied. Investigating whether performing core exercises with eyes closed and/or mental imagery affects proprioceptive adaptation is crucial for understanding motor control mechanisms and optimizing training strategies (Zhou et al., 2024; Ahmed and shosha, 2010). Investigating the effects of performing core exercises with eyes closed and without directed attention versus executing them with deliberate technique is essential to understanding the role of sensory and cognitive factors in motor control and neuromuscular adaptation (Huang et al., 2025; Nieto-Guisado et al., 2022). Core stabilization exercises are widely used in both athletic and rehabilitative contexts to enhance stability, strength, and injury prevention (Trampas et al., 2015; Skundric et al., 2021). Nonetheless, alterations in sensory input and attentional focus may modulate neuromuscular responses and thus impact training outcomes.

Motor imagery (MI)—the mental simulation of movement—has been shown to activate neural networks involved in actual movement execution, including the primary motor cortex, somatosensory regions, basal ganglia, and cerebellum (Wright et al., 2022; Çiftçi and Yılmaz, 2024). MI enhances motor learning and proprioceptive acuity, especially when integrated with physical practice. Thus, this study aimed to evaluate the effect of repeated maximal lumbar movements combined with MI on conscious lumbar proprioception in elite swimmers. We hypothesized that the group performing motor imagery in combination with lumbar movements would demonstrate greater improvements in proprioceptive accuracy than the group performing exercises with technical precision and breathing control alone, or the control group.

## 2 Materials and methods

### 2.1 Study design

**FIGURE 1:**
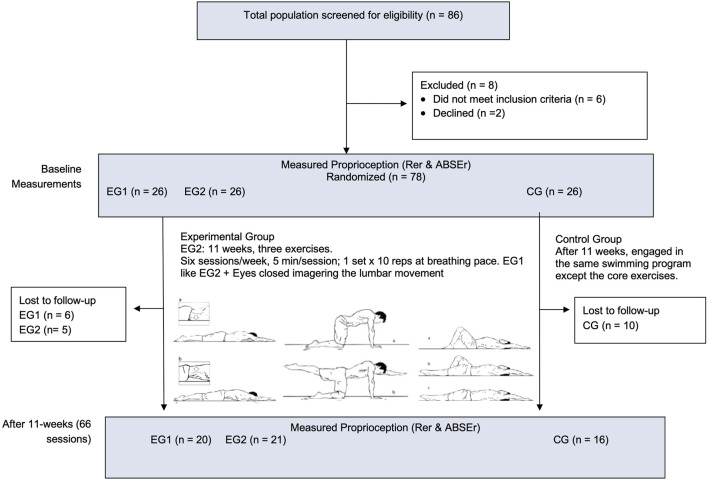
Flowchart and graphical methodological design. REr, Relative repositioning error; ABSEr, Absolute error; EG1, experimental group 1; EG2, experimental group 2; CG, control group.

We conducted a randomized clinical trial according to the CONSORT checklist (Eysenbach, 2013). The arms of this study and its flowchart are shown in Figure 1. Informed consent was obtained from all participants, and all procedures were conducted according to the Declaration of Helsinki. The research protocol was approved by the institutional Human Research Ethics Committee of Ramon Llull University, Spain (number 0000001DA, 17th November 2017). This study was registered on the Trial registration Current Controlled Trials website at www.ClinicalTrials.gov (accessed on 4th December 2024) (NCT06747702).

Due to the nature of the intervention, full participant blinding was not feasible; however, participants were not informed about group allocation or the specific hypotheses being tested. All participants were told that the study aimed to examine the effects of different training approaches on proprioception and motor performance, thus reducing expectation bias. The four coaches who supervised the physical training were unaware of the group assignment rationale and were not involved in outcome measurements or data analysis. Furthermore, all performance and proprioception assessments were conducted by independent assessors who were fully blinded to group allocation, ensuring objectivity in outcome evaluation.

### 2.2 Study population

A total of 78 professional swimmers were initially recruited and randomly assigned to three groups (EG1, EG2, and CG), with 26 participants per group. This sample size was determined through an *a priori* power analysis using G*Power 3.1(Bonn FRG, University of Bonn, Department of Psychology), assuming a large effect size (f = 0.4), α = 0.05, and a desired power (1−β) of 0.80 for repeated-measures ANOVA. Anticipating possible dropouts, we recruited a larger initial sample. Ultimately, 57 swimmers completed the study (Figure 1). A *post hoc* power analysis based on the observed partial η^2^ = 0.108 (f ≈ 0.35) revealed that the final sample size maintained sufficient statistical power (1−β = 0.81) to detect group × time interaction effects. They were recruited from the Spanish national swimming team. All participants met the inclusion criteria (Table 1) and were informed, in writing and verbally, about the procedures of this study prior to the assessment day.

**TABLE 1:** Inclusion criteria.

1. Part of the national Spanish swimming team
2. Minimum 4 years of experience at this high level
3. Did not suffer from any ailment or discomfort that would prevent him/her from competing, performing the exercises, or lumbar range of motion
4. Achieved an elite status and held international rankings in their respective age categories
5. Did not take medications throughout this study
6. Free of musculoskeletal injuries during the previous 3 months

After receiving detailed information, each participant signed an informed consent form, according with the World Medical Association’s Declaration of Helsinki (2024) (Resneck, 2025). The participant characteristics are shown in Table 2.

**TABLE 2:** Descriptive characteristics of the participants.

Age (yr), mean (SD)	
Males	20.2 (4.2)
Females	20.7 (3.3)
Gender, n female (%)	23 (40.3%)
Body mass Kg, mean (SD)	69.7 (10.3)
Height, cm, mean (SD)	177.8 (7.5)
Professional swimming experience (years) mean (SD)	8.7 (4.4)
Level, n (%)	
Olympic	33 (57.8%)
International	13 (22.8%)
National	11 (19.2%)
Main swimming style n (%)	
Freestyle	18 (31.5%)
Breaststroke	14 (24.5%)
Butterfly	9 (15.7%)
Backstroke	8 (14.0%)
Individual medley	8 (14.0%)
Main competition distance n (%)	
50–100	20 (35.1%)
200–400	31 (54.4%)
800–1,500	6 (10.5%)

### 2.3 Procedures

All participants were recruited from two training camps of the Spanish swimming national team. The swimmers who agreed to participate were interviewed to collect descriptive data (Table 2) and tested for the first time at their training facilities. The participants were randomly divided using the online randomization software Research Randomizer (randomizer.org, accessed on 2nd October 2024) into EG1, EG2, and CG (Figure 1).

**FIGURE 2:**
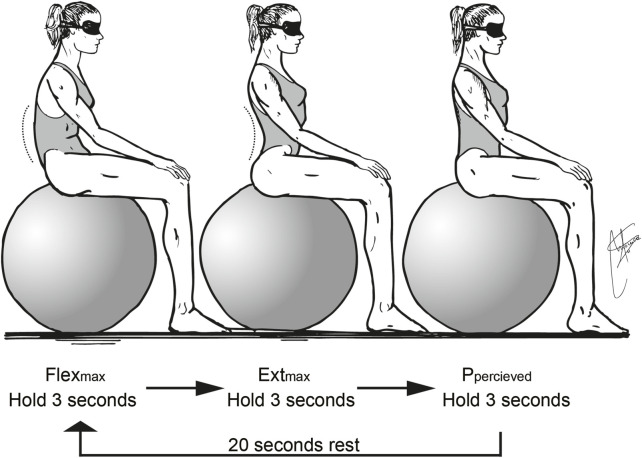
Details of required lumbar extension during inhalation and return to a lumbar aligned position during exhalation.

After performing the baseline measurements (pre-test), both experimental groups (EG1 and EG2) received detailed instruction on the technical execution of the exercises and how to use controlled breathing to structure repetitions. Participants were instructed to perform maximal lumbar extension during inhalation, followed by a return to a neutral, aligned position during exhalation (see Figure 2). In addition, EG1 received specific training in motor imagery focused on lumbopelvic movements. This included an explanation of the anatomical and biomechanical principles of the lumbar spine during sagittal plane motion, supported by the visualization of a 3D model of lumbopelvic kinematics. A dedicated 2-h session was conducted to introduce and guide participants in the use of motor imagery.

During the exercises, EG1 participants were also instructed to close their eyes and concentrate on the perceived movement of their lumbar spine, actively engaging in imagery-based rehearsal to enhance proprioceptive awareness.

The imagery protocol focused on kinesthetic imagery, encouraging participants to mentally simulate the internal sensations associated with lumbopelvic movement and spinal alignment. A first-person internal perspective was used to reinforce the motor simulation of proprioceptive sensations during lumbar movement.

The imagery training was structured using guided verbal instructions. The script included three phases:

• Preparation (e.g., “Assume the exercise position and take a deep breath”).
• Imagery cue (e.g., “Feel your lumbar spine actively extend as you inhale”).
• Alignment cue (e.g., “While exhaling, visualise and feel the lumbar region aligning with the body’s axis”).

Four national coaches were instructed to guide and control the development of the intervention. The intervention protocol involved performing three core exercises in the sagittal plane, synchronized with a controlled breathing pace.

### 2.4 Training and motor imagery protocol

Each exercise was executed for a single set of ten breaths at a natural respiratory rate (Figure 1). A 30s rest period was allocated between exercises to ensure recovery and maintain movement quality. Given the breathing pace of one breath every 5–6 s, the total duration of the intervention ranged from 4 to 5 min. To minimize potential nervous system fatigue and maximize neuromuscular activation, the exercises were strategically incorporated as the initial component of the swimmers*’* specific warm-up routine.

Exercise 1: From a prone position, with the arms extended overhead in the streamline position used in undulatory swimming and the entire body fully extended, the participant inhales while actively arching the lumbar spine as much as possible. During exhalation, the goal is to progressively align the lumbar spine with the body’s longitudinal axis to the greatest extent possible.

Exercise 2: From an all-fours position, with the hands aligned under the shoulders and the knees under the hips, inhale while arching the lumbar spine as much as possible. During exhalation, aim to align the lumbar spine with the body’s longitudinal axis while simultaneously extending one arm overhead and the opposite lower limb, reaching through the full length of the body to maximize longitudinal extension.

Exercise 3: From a supine position, with the arms extended overhead in the streamline position used in undulatory swimming and the legs bent at the knees with the feet hip-width apart, inhale while actively arching the lumbar spine as much as possible. During exhalation, aim to align the lumbar spine with the body’s longitudinal axis while simultaneously extending the knees to bring the entire body into the underwater streamline position.

Motor imagery protocol:

The motor imagery (MI) protocol was designed and reported following the *Guidelines for Reporting Action Simulation Studies* (GRASS; Moreno-Verdú et al., 2024), ensuring methodological transparency and reproducibility.

Imagery was performed concurrently with each repetition, lasting 5–6 s, and was repeated 30 times per session (10 per exercise), 3 times per week for 3 weeks. To enhance the ecological validity and neural activation associated with motor imagery (MI), the intervention was designed following the PETTLEP model (Physical, Environment, Task, Timing, Learning, Emotion, and Perspective), as recommended by Holmes and Collins (2001) and recently applied in sports science literature (Çiftçi and Yılmaz, 2024). Participants performed the imagery in the same physical position (e.g., standing, prone, or seated) as required by the actual execution of each specific exercise to match motor and postural congruence (Physical). All MI sessions took place in the same environment (training room or lab setting) as the physical practice, ensuring consistent sensory cues and reducing context-dependent variability (Environment). The task being imagined was identical in content and structure to the physical exercise, including muscle recruitment patterns and joint movements specific to lumbopelvic stabilisation tasks (Task). Imagery was conducted in real time (i.e., the duration of each imagined trial mirrored the temporal characteristics of actual performance), preserving the motor timing and internal pacing of the movement (Timing). Instructions were progressively adjusted to reflect learning stage progression and individual familiarity with the exercise, allowing participants to refine imagery content as their technical execution improved (Learning). Athletes were encouraged to evoke emotional states associated with competitive performance (e.g., confidence, focus), as emotional congruence has been shown to enhance MI vividness and effectiveness (Emotion). Finally, imagery was performed from a first-person visual and kinaesthetic perspective, reinforcing internal simulation and activating sensorimotor representations linked to the execution of sport-specific skills (Perspective).

None of the participants in EG1 had prior experience with structured imagery training. A 2-h introductory session was conducted at the beginning of the intervention to provide instruction, demonstration, and individual feedback on motor imagery techniques.

### 2.5 Testing protocol

Lumbar joint position sense (JPS) was assessed using a twin-axis electrogoniometer (Transducer TSD130A, Biopac Systems, Inc., Goleta, California, United States), which was integrated with a computer and Acknowledge 3.0.9 software (Biopac Systems, Inc., Goleta, California, United States) Lumbar flexion, extension, and total ROM degrees were measured. The equipment was calibrated prior to each testing day to determine the 0° and 90° of each frontal and sagittal plane, but only the sagittal data were analyzed as is the plane of movement of undulatory underwater swimming (UUS). The cranial arm of the goniometer was placed over the D11 and D12 spinal processes, while the lower arm was placed over the sacrum. Therefore, flexion movements were associated with positive degrees, and extension movements were associated with negative degrees. Lumbar ROM scores were obtained by summing the mean flexion degrees and the mean absolute extension degrees collected in each trial.

The computer was calibrated with a sample rate of 500 Hz. A manual chronometer (Namaste^©^ model 898, Gran Canarias, Spain) was used to identify the interval in seconds over which the subjects maintained each position at the recorded degrees.

**FIGURE 3:**
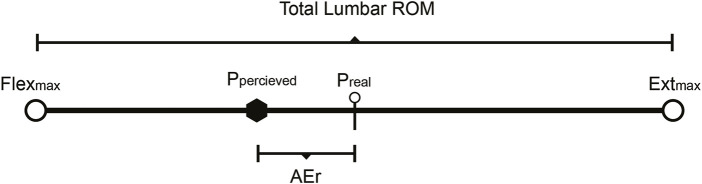
Testing procedures: electrogoniometer placement (D11 = 11th dorsal vertebra, D12 = 12th dorsal vertebra, L3 = 3rd lumbar vertebra, L4 = 4th lumbar vertebra, L5 = 5th lumbar vertebra, S1 = 1st sacral vertebra, S2 = 2nd sacral vertebra) testing position.

Different Swiss balls (Gymnic Plus Stability physioballs, TMI, Inc., Osoppo, Údine, Italy), ranging in diameter from 55 to 90 cm, were used to ensure a correct seated body position, at 90 degrees of the hips and knee flexion, with the feet separated at hip height to increase seated stability. The ball inflation was checked at 3 bars between tests to ensure that the diameter remained stable. We used three sizes of Swiss balls during the evaluation: 55 cm for subjects between 1.60 and 1.70 m tall, 65 cm for subjects between 1.71 and 1.80 m tall, and 90 cm for subjects between 1.81 m and 1.90 m tall (Figure 3).

The Swiss ball height was standardized across participants by selecting one of three predefined ball sizes (55, 65, or 75 cm) to ensure approximately 90° of hip and knee flexion. This adjustment followed a strict protocol based on participant height and was recorded for each individual to minimize variability. Intra-rater reliability was calculated using intraclass correlation coefficients (ICCs), yielding ICC = 0.91 (95% CI: 0.83–0.96), while inter-rater reliability was ICC = 0.88 (95% CI: 0.79–0.94), indicating excellent reliability according to established criteria (Solana-Tramunt et al., 2019b). All electrogoniometric measurements were performed by trained evaluators who were blinded to group allocation.

All tests were completed between 2 and 5 p.m. by the same primary investigator to minimize fluctuations in circadian lumbar ROM (Solana-Tramunt et al., 2019b). Lumbar ROM scores were obtained by summing the mean flexion degrees and the mean absolute extension degrees collected in each trial. ABSEr degrees were obtained based on the difference in degrees of the real mathematical mid position (Preal) and the perceived mid position (Pperceived) (Figure 3). Relative Errors percentages (REr) were obtained dividing AEr by the total ROM and multiplying it by 100 (AEr/ROM * 100).

### 2.6 Statistical analyses

IBM SPSS Statistics for Windows, Version 28.0 (IBM Corp., Armonk, NY, United States) was used for statistical analysis. The descriptive data of the variables are presented as mean (SD). The distribution of the variables was verified using the Kolmogorov–Smirnov test. A repeated measures MANOVA was conducted to evaluate the effects of Group (EG1, EG2, CG) and Time (Test, Retest) on two dependent variables: Relative Error (REr) and Absolute Error (ABSEr). The model included the main effects of Group and Time, as well as the Group × Time interaction. Prior to analysis, assumptions of normality, homogeneity of covariance matrices (Box’s M test), and absence of multicollinearity (correlations <0.80) were tested and met.

Significant multivariate effects were followed by univariate analyses for each dependent variable. When univariate main or interaction effects were significant, Bonferroni-adjusted pairwise comparisons were conducted to identify specific group differences. The significance level was set at p < 0.05.

Effect sizes were reported using partial eta squared (η^2^p) for MANOVA and ANOVA effects, with thresholds interpreted as small (0.01), medium (0.06), and large (0.14). Additionally, Cohen’s d was calculated for within-group pre-to post-intervention changes to assess the magnitude of individual-level improvements. Cohen’s d was interpreted as small (0.2), moderate (0.5), and large (0.8).

## 3 Results

**FIGURE 4:**
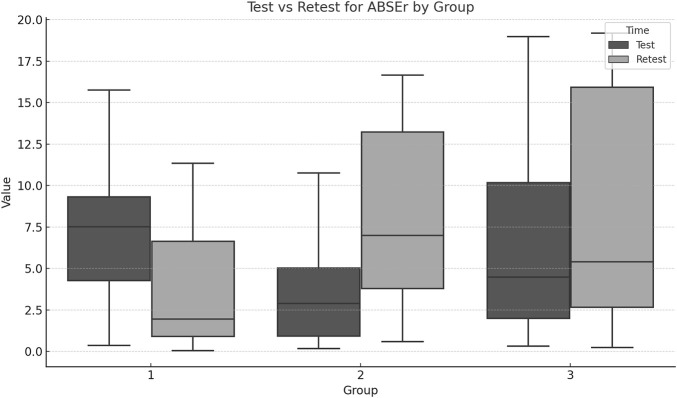
Boxplots for each group ABSEr across Test and Retest moments. Erase **(A)** and **(B)** labels.

**FIGURE 5:**
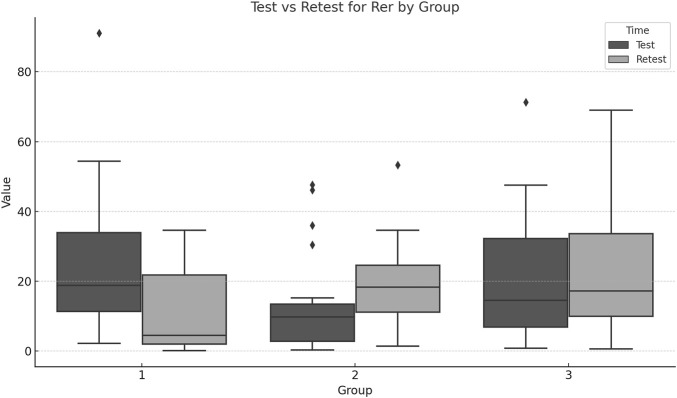
Boxplots for each group REr across Test and Retest moments.

Significant group differences were found for Retest_REr (F = 6.75, p = 0.012) and Retest_ABSEr (F = 7.34, *p* = 0.009). The repeated measures MANOVA showed significant differences in the multivariate profiles across groups and over time. Comparing Test and Retest values for each group indicates whether performance or the measured outcome changed significantly over time (Figures 4, 5; Table 3).

**TABLE 3:** Rer and ABSEr test-retest results.

Group	RerTest	Rer retest	*d* (95% CI)	ABSErTest	ABSEr retest	*d* (95% CI)
EG1	25.6	10.3.5	0.9	7.2	3.5*	1.2
	(21.0)	(11.0)	(0.44–1.3)	(4.0)	(3.6)	(0.7–1.7)
EG2	13.1	18.9	−0.5	3.5	8.0*	−1.1
	(14.4)	(12.4)	(−1.0 to 0.1)	(3.0)	(5.1)	(−1.6 to 0.7)
CG	20.0	22.3	−0.2	6.9	8.3	−0.3
	(19.5)	(19.1)	(−0.8 to 0.2)	(6.6)	(7.3)	(−0.8 to 0.2)

EG1 achieved large positive effect sizes for both REr (d = 0.91) and ABSEr (d = 1.24), with tight confidence intervals, indicating meaningful improvements in lumbar proprioception. EG2 showed moderate to large negative effect sizes (REr d = −0.56, ABSEr d = −1.17).

## 4 Discussion

This study aimed to examine the effects of repeated maximal lumbar movement, performed with eyes closed and accompanied by motor imagery (MI), on lumbar proprioception in elite swimmers. The results demonstrate that only the experimental group using MI (EG1) exhibited significant improvements in both relative repositioning error (REr) and absolute error (ABSEr), as evidenced by Cohen’s d effect sizes. In contrast, the control group (CG) and the experimental group without imagery (EG2) showed small or negligible changes.

Specifically, core exercises mimicking undulatory underwater swimming (UUS) improved lumbar proprioception only when paired with directed internal focus and visual deprivation. EG1 participants achieved better joint position sense by integrating conscious attentional control and kinesthetic visualization. This suggests that technical execution alone, as in EG2, is insufficient to enhance conscious proprioceptive awareness.

A key differentiator was the inclusion of motor imagery in EG1. Participants received anatomical instruction prior to training and were guided to visualize lumbar movements while performing them. This combination of motor execution and imagery likely enhanced sensorimotor processing by activating shared neural pathways, as supported by neuroimaging studies showing MI-related activation in motor-related cortical areas (Mizuguchi et al., 2012; Smith et al., 1985). Additionally, restricting visual input may have amplified reliance on somatosensory feedback (Nieto-Guisado et al., 2022; Nieto-Guisado et al., 2024), further improving proprioceptive acuity. However, we acknowledge that while this explanation aligns with previous findings, claims regarding cortical sensory representation changes remain hypothetical. No neurophysiological measures were taken to support such mechanisms in this study. Thus, while plausible, these interpretations should be viewed cautiously and considered for future investigation.

In contrast, EG2, which performed the same physical exercises without MI, showed a deterioration in proprioceptive performance. Negative effect sizes in both REr and ABSEr suggest that physical training without internal focus or imagery may not only be insufficient but potentially counterproductive in highly trained athletes. These finding challenges prior evidence supporting generalized core training for proprioceptive improvement and highlights the importance of cognitive engagement and attentional focus (Behm and Chaouachi, 2011). The exercise protocol instructed participants to actively reach their maximal lumbar extension during inhalation and then transition to a streamlined spinal alignment during exhalation. This sequence aimed to dynamically stretch the abdominal musculature while eliciting contraction of the lumbar erector spinae. Importantly, the protocol did not involve stretching of the posterior lumbar muscles, as the movement concluded in a neutral spinal alignment, thereby explaining that the exercises were designed to improve the ability to detect the lumbar position where the first pull for UUS is performed. EG1 participants performed the same exercises as EG2 but with eyes closed and directed attentional focus on lumbar movement. This conscious control of movement may have increased proprioceptive activity, as evidence suggests that proprioceptive acuity is enhanced when visual input is restricted and attention is focused on internal sensory feedback.

Moreover, participants in EG1 received instruction in lumbar anatomy and biomechanics prior to the intervention, which was intended to enhance their capacity for motor imagery. Motor imagery, commonly employed in athletic training to optimize performance, activates specific neural pathways involved in motor control (Mizuguchi et al., 2012; Short et al., 2005). Neuroimaging studies have shown that brain activity during motor imagery closely mirrors that of actual movement, particularly when the sensory context mimics real execution. In this study, cognitive imagery was integrated into the exercise sessions, as participants were instructed to mentally visualize lumbar movements while simultaneously performing them.

An unexpected finding of this study was the deterioration in proprioceptive accuracy observed in EG2, as indicated by negative effect sizes in both relative repositioning error (REr) and absolute error (ABSEr). Although all groups completed the same core training program, only EG1—which combined physical training with motor imagery (MI)—demonstrated significant improvements in joint position sense. In contrast, EG2 exhibited reduced proprioceptive performance, a result that contradicts the generally positive impact of core stability training on sensorimotor control reported in previous literature. Several factors may explain this counterintuitive outcome. First, the absence of MI in EG2 may have limited the central integration of proprioceptive information. Motor imagery has been shown to enhance neural activation in sensorimotor areas involved in proprioception and joint position control (Lebon et al., 2018). Without this internal simulation, EG2 participants may have relied more heavily on mechanical execution, lacking the attentional focus and kinaesthetic refinement provided by MI. Second, while the training load was equal across groups, EG2 may have experienced greater cognitive fatigue or disengagement due to the repetitive nature of the tasks, potentially interfering with central proprioceptive processing. Previous research highlights the importance of attentional focus and cognitive engagement in motor learning and proprioceptive adaptation (Wulf and Lewthwaite, 2016).

The brief core workout was integrated at the beginning of each morning warm-up session to maintain the ecological validity of the training routine and to avoid disrupting the athletes’ habitual practice. This timing also ensured that exercises were performed in a neuromuscular fresh state, as central nervous system (CNS) fatigue has been shown to impair motor control and proprioceptive acuity (Lee et al., 2003). To enhance adherence over the 11-week intervention (six sessions per week), the protocol was limited to three core exercises, performed in a single set of 10 repetitions. This minimal approach aimed to reduce participant burden and mitigate dropout risk. Despite these efforts, the control group (CG) lost 10 participants and the experimental group (EG) lost 11. In elite sport environments, preserving the ecological validity of interventions while designing efficient, time-conscious workouts is crucial for maintaining athlete engagement and aligning with the goals of coaches and performance staff.

It is widely accepted that core training programs should target the same muscle chains and movement patterns used in the sport-specific technique to ensure high transferability to performance (Lederman, 2010). However, research examining sport-specific core training for swimming remains limited. Most studies rely on generalized core exercises, which may be included in the conditioning routines of athletes from any discipline, lacking specificity to the biomechanical and neuromuscular demands of swimming. For instance, one study investigated the effects of a 6-week generalized core training program on national-level swimmers. Despite improvements in individual swimming variables being statistically non-significant, the experimental group improved their 50-m front crawl performance by 1.2%, compared to 0.7% in the control group (Khiyami et al., 2022; Ji et al., 2021; Kurt et al., 2023). Another study implemented a 12-week dry-land core training program divided into three progressive phases: stabilization (e.g., bridges, planks, bird dogs), muscular power (e.g., unilateral deadlifts, squats, rows), and power endurance (e.g., medicine ball slams, one-arm snatches, chops). This program produced significant improvements in anaerobic power, core stability, upper body muscular endurance, and overall swimming performance. However, the exercises remained non-specific to swimming technique (Karpiński et al., 2020). Similarly, a 6-week non-functional core training program applied three times weekly alongside regular swim training resulted in significant improvements in freestyle swimming performance and various core muscle properties—including contractility, excitability, extensibility, and elasticity—among young swimmers. Notably, this study’s results are limited to adolescent recreational swimmers (aged 13 ± 2 years) with varying maturational stages, restricting generalizability to elite or older populations (Ji et al., 2021).

This study was specifically designed to target lumbar movements relevant to undulatory underwater swimming (UUS), with exercises replicating the neuromuscular synergies involved in three key phases of UUS: the transition from the upward to the downward kick, the downward kick, and the upward kick. UUS plays a pivotal role in starts and turns across all strokes—especially in backstroke and butterfly—where optimal wave amplitude and frequency are critically dependent on lumbar flexibility and motor control (Ruiz-Navarro et al., 2022; Matsuura et al., 2020).

Recent literature underscores the relationship between increased lumbar ROM and improved UUS performance among elite swimmers. A systematic review by Veiga et al. (2022) reported that a greater ROM in the lower trunk during undulatory movement correlates with higher forward swimming velocities during the UUS phase. Nevertheless, the increases on ROM must be accompanied by an increase in the ability to control the movement, as the coordination of different muscle actions across the UUS is crucial for developing efficient movement patterns and increasing performance.

To ensure that participant attrition did not introduce bias in the group composition, a *post hoc* analysis was conducted to assess changes in stroke specialization distribution across the control group (CG) and experimental group (EG). The analysis confirmed that the proportions of swimmers by stroke remained balanced across groups, despite participant dropout. This maintained distribution minimized the potential for confounding effects due to stroke-specific movement profiles. Moreover, as the study’s primary objective was to assess changes in lumbar proprioception rather than stroke-specific adaptations, balanced representation across swimming styles was essential for ensuring the internal validity and generalizability of the findings.

All three exercises implemented in the EG1 and EG2 required participants to achieve maximal lumbar extension during inhalation, emphasizing activation of the erector spinae (ES) and multifidus (MF), and to return to a streamlined lumbar alignment during exhalation, engaging the rectus abdominis (RA), internal oblique (IO), and transversus abdominis (TrA) muscles. These exercises were performed in prone, quadruped (four-point kneeling), and supine positions. Exercises 1 and 2 placed greater demand on the ES and MF due to the gravitational resistance encountered during anterior pelvic tilt and lumbar extension. In contrast, Exercise three primarily challenged the RA and IO, as gravity opposed the posterior pelvic tilt and lumbar flexion movements. In the context of undulatory underwater swimming (UUS) among elite swimmers, both the upward and downward kicks are closely coordinated with the trunk musculature involved in pelvic anterior–posterior tilting (Ruiz-Navarro et al., 2022). Furthermore, effective dolphin kicking requires alternating contractions of the trunk, thigh, and leg flexor and extensor muscles to generate propulsive wave motion (Veiga et al., 2022). Functional movement patterns, therefore, play a critical role in achieving high levels of performance, particularly in swimming, where optimal spinal and pelvic kinematics contribute directly to propulsion efficiency.

It is widely recognized that defining and assessing an athlete’s functional movement relative to their specific sport technique is essential for both performance optimization and injury prevention.

Although it was not the primary aim of this study, we intentionally synchronized participants’ breathing patterns with their lumbar movements. Specifically, participants were instructed to inhale during lumbar extension and exhale while returning to the streamlined lumbar position. This breathing pattern was proposed to enhance the engagement of core musculature, as previous research has demonstrated that inspiratory efforts recruit the erector spinae (ES) and multifidus (MF), whereas voluntary expiration predominantly activates the transversus abdominis (TrA), rectus abdominis (RA), internal obliques (IO), and both major and minor oblique muscles (Hodges et al., 2003). Moreover, multiple studies have suggested that consciously controlling breathing during exercise can improve pelvic positioning and lumbar alignment by reinforcing neuromuscular coordination between respiratory and postural muscles (Hodges et al., 2003). The deliberate inclusion of forced expiration during lumbar flexion aimed to further activate the TrA, a deep abdominal muscle recognized for its key role in enhancing core stiffness and stability. Given its capacity to increase intra-abdominal pressure and reduce trunk perturbation, TrA activation is considered essential in minimizing hydrodynamic drag during undulatory underwater swimming (UUS) (Matsuura et al., 2020; Veiga et al., 2022; Connaboy et al., 2010).

Regarding the testing procedures and instruments, lumbar range of motion (ROM) was assessed using an electrogoniometer while participants were seated on a Swiss ball. This seated position was chosen to allow unrestricted movement of the lumbar spine in the sagittal plane while ensuring postural stability. Participants maintained both feet flat on the floor, hip-width apart, which provided a balanced and comfortable position that enabled maximal flexion and extension without compromising alignment or inducing discomfort.

One of the primary limitations of this study was the high attrition rate (37%), with 21 participants not completing the intervention or retest assessments. Although dropout was largely due to training and competition schedules, this reduction in sample size may have compromised statistical power and increased the risk of bias in group comparability. To address this, we conducted a *post hoc* analysis confirming that stroke specialization remained balanced across groups, helping to mitigate confounding from stroke-specific biomechanical profiles. However, without intention-to-treat (ITT) or imputation strategies, the findings may be subject to attrition bias. Future studies should incorporate retention-enhancing protocols (e.g., shorter interventions, digital reminders, structured incentives) and consider sensitivity analyses to validate the robustness of observed effects. Furthermore, the study focused primarily on lumbar joint position sense without measuring actual swimming performance (e.g., UUS velocity, turn times). This limits our ability to directly link proprioceptive improvements to competitive outcomes. Future research should integrate biomechanical and performance-based metrics to better evaluate the translational impact of sensorimotor training.

The present findings suggest that integrating motor imagery with sport-specific core exercises can significantly enhance lumbar proprioception in elite swimmers. These improvements may contribute to better segmental control, refined pelvic tilting, and potentially improved UUS efficiency-particularly in strokes requiring high degrees of trunk undulation. Incorporating short, targeted proprioceptive routines into dryland training could offer a time-efficient strategy for enhancing neuromuscular coordination and reducing the risk of overuse lumbar injuries in swimmers. Coaches and practitioners are encouraged to implement attentional strategies and imagery-based techniques in core training to maximize both athletic performance and movement quality in the aquatic environment.

## 5 Conclusion

This study demonstrates that physical training alone may not be sufficient to enhance proprioceptive acuity in elite swimmers. Only the group combining core exercises with motor imagery (MI) and visual deprivation showed significant improvements in lumbar joint position sense. In contrast, performing the same exercises with technical focus alone led to reduced proprioceptive accuracy, underscoring the role of cognitive engagement in sensorimotor adaptation.

The addition of MI likely enhanced neuromuscular control by promoting internal focus and cortical sensory integration. The improvement in proprioceptive accuracy has practical implications for optimizing movement quality and preventing overuse injuries, especially in disciplines reliant on undulatory underwater swimming (UUS).

These findings support the integration of cognitive strategies, such as MI and internal cueing, into core training routines. Coaches and practitioners are encouraged to adopt brief, targeted protocols combining physical execution with mental rehearsal to enhance proprioception, refine motor control, and promote long-term spinal health in elite swimmers.

## Data Availability

The raw data supporting the conclusions of this article will be made available by the authors, without undue reservation.
